# Neural presbycusis at ultra-high frequency in aged common marmosets and rhesus monkeys

**DOI:** 10.18632/aging.202936

**Published:** 2021-04-27

**Authors:** Zhuoer Sun, Zhenzhe Cheng, Neng Gong, Zhen Xu, Chenxi Jin, Hao Wu, Yong Tao

**Affiliations:** 1 Department of Otolaryngology-Head and Neck Surgery, Shanghai Ninth People’s Hospital, Shanghai Jiaotong University School of Medicine, Shanghai 200011, P.R. China; 2 Ear Institute, Shanghai Jiaotong University School of Medicine, Shanghai 200011, P.R. China; 3 Shanghai Key Laboratory of Translation Medicine on Ear and Nose Disease, Shanghai 200011, P.R. China; 4 Institute of Neuroscience, Key Laboratory of Primate Neurobiology, CAS Center for Excellence in Brain Science and Intelligence Technology, Chinese Academy of Sciences, Shanghai 200031, P.R. China; 5 University of Chinese Academy of Sciences, Beijing 100049, P.R. China

**Keywords:** age-related hearing loss, non-human primate, deafness, neural degeneration

## Abstract

The aging of the population and environmental noise have contributed to high rates of presbycusis, also known as age-related hearing loss (ARHL). Because mice have a relatively short life span, murine models have not been suitable for determining the mechanism of presbycusis development and methods of diagnosis. Although the common marmoset, a non-human primate (NHP), is an ideal animal model for studying age-related diseases, its auditory spectrum has not been systematically studied. Auditory brainstem responses (ABRs) from 38 marmosets of different ages demonstrated that auditory function correlated with age. Hearing loss in geriatric common marmosets started at ultra-high frequency (>16 kHz), then extended to lower frequencies. Despite age-related deterioration of ABR threshold and amplitude in marmosets, outer hair cell (OHC) function remained stable at all ages. Spiral ganglion neurons (SGNs), which are the first auditory neurons in the auditory system, were found to degenerate distinctly in aged common marmosets, indicating that neural degeneration caused presbycusis in these animals. Similarly, age-associated ABR deterioration without loss of OHC function was observed in another NHP, rhesus monkeys. Audiometry results from these two species of NHP suggested that NHPs were ideal for studying ARHL and that neural presbycusis at high frequency may be prevalent in primates.

## INTRODUCTION

Age-related hearing loss (ARHL), or presbycusis, is one of the most common deteriorations associated with aging. The National Institute on Deafness has estimated that 33% of people over age 65 years have significant hearing loss. ARHL, however, presents with various symptoms in elderly people [1], including reduced hearing sensitivity and understanding of speech in noisy environments, slowed central processing of acoustic information, and impaired localization of sound sources. Presbycusis has a deleterious effect on communications and can lead to isolation, depression, and dementia [2]. Moreover, deterioration in auditory function synchronously affects cognitive ability [3], making ARHL a more systematic and widespread health problem.

ARHL is also known as sensorineural hearing loss because the dysfunction arises in the cochleae, in which sound-induced vibrations are transduced into cochlear neurons by electrical signals. The etiology of ARHL is multifactorial [4–7], including sensory, neural, metabolic and cochlear conduction causes [8]. Clinically detected presbycusis is not always a specific type but may be mixtures of pathologic types [8].

Due to the complexity of presbycusis in humans, much knowledge about ARHL has been derived from animal models. Studies in animals have shown the degeneration of the stria vascularis, the sensorineural epithelium and neurons of the central auditory pathways in aged cochleae [9]. Synaptic loss in aged mice was recently shown to be unaccompanied by hair cell damage [10–12], and age-related loss of cochlear synapses was found to precede both hair cell loss and threshold elevation [6, 13]. Although primary cochlear neuron degeneration was found in cadavers of elderly people [13], this has not been validated in other species.

Rodent models are widely used to study the genetics of ARHL, but their shorter life span and broader auditory spectrum limit their use in ARHL research. Non-human primates (NHPs) are closer to humans phylogenetically, especially in modeling sensory and neurological defects [14], and have been utilized as models of aging in humans [15], suggesting that NHPs may constitute better models of ARHL than mice. Gene therapy was able to restore hearing loss in deafness mice, and NHPs was essential model for gene therapy translation [16–20].

NHPs include Old World monkeys, such as rhesus monkeys, and New World monkeys, such as marmosets, which have advantages in many areas of biomedical research [21]. Rhesus monkeys, which share 92% genetic homology with human [22], have a relatively long lifespan, with an average of 25 years and a maximum of 40 years in captivity. Common marmosets (*Callithrix jacchus*) may also be a valuable NHP for modeling human diseases [23], as their age-related pathology is similar to humans, including cancer, diabetes, chronic renal disease and amyloidosis [24]. Common marmosets give birth to twins, are of relatively small size, develop rapidly and have a relatively short lifespan, thus yielding experimental results more rapidly and at lower cost than rhesus monkeys [25].

Audiograms of rhesus monkeys taken over a 3-year period showed evidence of age-related presbycusis in these animals [26]. Aging in these animals was strongly associated with decreasing ABR sensitivity and extensive changes across multiple cochlear pathologies [27]. Aged rhesus monkeys also showed decreased cochlear and neural function, including smaller ABR peak amplitudes and significantly longer ABR peak latencies [27]. Distortion product otoacoustic emissions (DPOAE), generated by cochlear outer hair cells (OHC) in response to two tones close in frequency, was also observed in aged rhesus monkeys [27]. Less is known, however, about the characteristics of audiograms in aging marmosets. An assessment of brainstem auditory evoked potentials (BAEPs) in aging marmosets showed that BAEP latencies were apparently sensitive indicators of early age-related hearing impairment [28]. Recent measurements of the ability of marmosets to distinguish among pure tone frequencies across a broad frequency range showed that the highest concentration of spectral energy was at frequencies of 3.5-14 kHz [29]. Moreover, cochlear pathology was assessed in marmosets of different ages [30]. Few studies, however, have assessed differences among wide-ranging frequencies, which contain abundant and precise information about hearing. To our knowledge, little is known about the auditory and histologic characteristics of aging marmosets.

Clinical pathophysiology has indicated that a loss of threshold sensitivity at high frequencies is the first indicator of ARHL [2, 31]. Although high frequency hearing loss has been detected in nearly 2-year-old gerbils [32] and mice [33], the relationship between this hearing loss and ARHL in humans was unclear due to the hearing frequency gap between humans and rodents. Given that the auditory sensation frequency range is similar in NHPs and humans, high frequency audiometry (>8 kHz) in NHPs may assist in research on ARHL in humans. Because ARHL is considered accumulated noise induced hearing impairment, animals of relatively long life span are essential for research on presbycusis. The present study therefore focused on the correlation between age and high-frequency audiometry in two types of NHP, marmosets and rhesus monkeys.

Auditory neuropathy or dys-synchrony describes a type of hearing impairment in which OHC function is normal but afferent neural conduction in the auditory pathway is disordered [34]. Neural and/or behavioral audiograms from geriatric humans and animals have shown poor correlation with OHC function [35]. Cochlear inner hair cells (IHCs), synapses between IHCs and auditory nerve terminals, auditory nerve abnormalities, and myelin disorders may contribute to neural presbycusis, but the evidence remains unconvincing [36]. In addition to hearing loss, older humans have other auditory problems, suggesting that neural presbycusis could explain ARHL-associated changes in psychophysical performance with suprathreshold stimuli [35], which are difficult to evaluate in rodents. NHPs may be appropriate models for ARHL research, but little is unknown about auditory neuropathy in aging animals.

The present study compared auditory functions of marmosets of different ages at various frequency ranges, and observed age-related ABR sensitivity at ultra-high frequencies without DPOAE alterations. Examination of cochlear pathology in younger and older marmosets showed that loss of SGN was the main cause of ARHL in marmosets. In addition, this study confirmed that auditory dys-synchrony was present in rhesus monkeys, another species of NHP.

## RESULTS

### ABR thresholds at ultra-high frequencies in geriatric marmosets

ABRs to tones were recorded in 38 marmosets aged 2 years and 1 month to 10 years and 5 months. Marmosets age at a rate approximately seven times faster than humans, so these animals were roughly equivalent humans aged 14 to 70 years. Because auditory sensitivity in these animals became weaker at frequencies >32 kHz, hearing at up to 32 kHz was tested in these animals [28].

**Figure 1:**
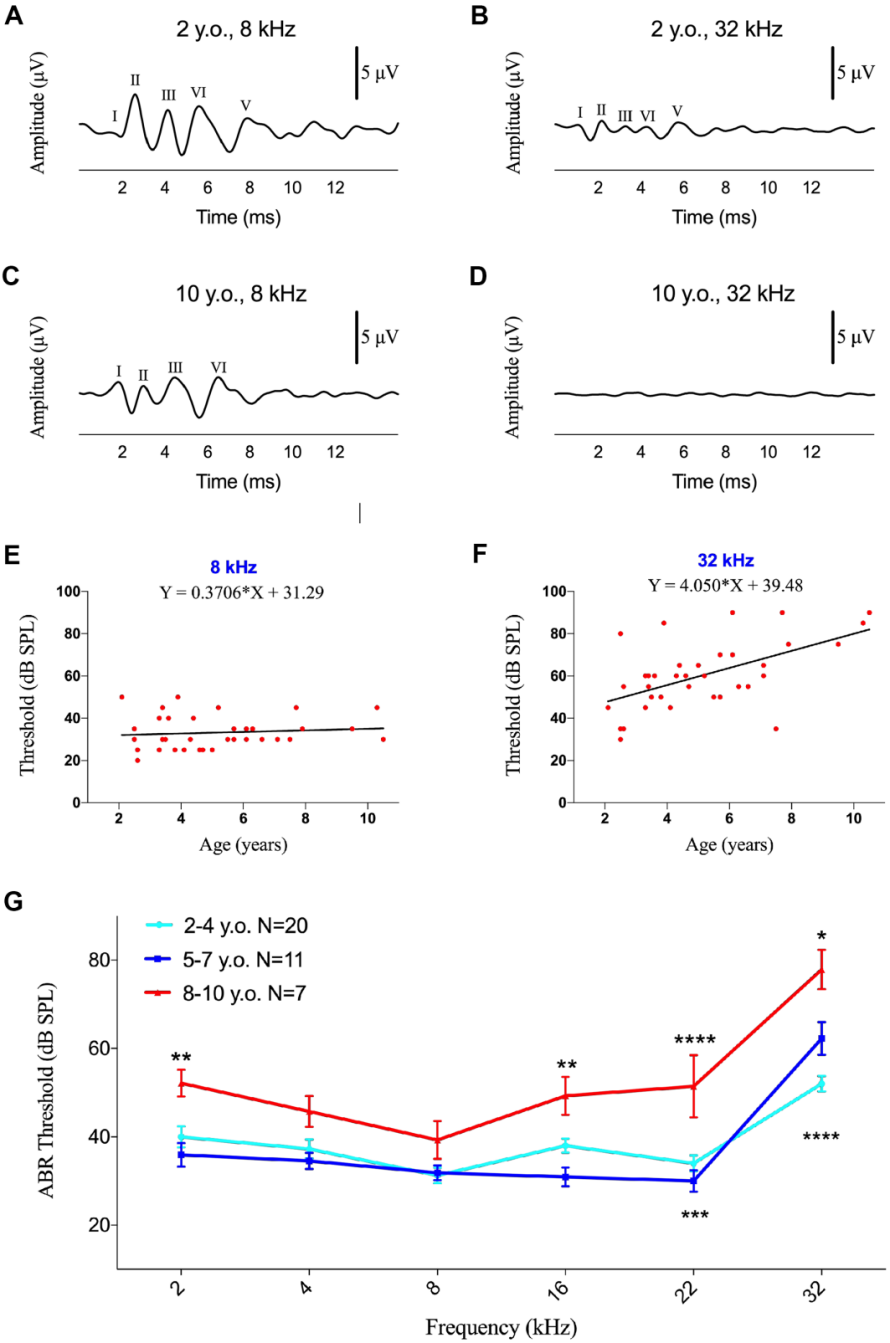
**Effects of age on ABR thresholds in marmosets.** (**A**–**D**) Examples of ABR waveforms of common marmosets aged 2 and 10 years following 80 dB SPL tone bursts at frequencies of 8 and 32 kHz. (**E**, **F**) Scatter plots and corresponding regression lines and regression equations for the relationships between ABR thresholds (dB SPL) of 36 individual marmosets and age (years) at frequencies of tone burst stimuli of 8 kHz (**E**) R-squared linear=0.01168, P=0.5304) and 32 kHz (**F**) R-squared linear=0.3172, P=0.0003). (**G**) Relationships between ages and auditory thresholds determined by ABR. Data are presented as mean ± SEM. **P < 0.01; ****P < 0.0001 by two-way ANOVA for significant differences in ABR thresholds between 2-4-year-old and 8-10-year-old marmosets; *P < 0.05, ***P < 0.001 by two-way ANOVA for significant differences in ABR thresholds between 5-7-year-old and 8-10-year-old animals.

The ABR waveform during the first 10ms after a stimulus consisted of five positive waves (I-V), with five to six positive waves detected at high sound pressure (Figure 1A–1D). Not all of these components were detected in all animals. ABR morphology did not differ significantly between male and female marmosets. Comparisons of the size and latency of the peaks in each trace from top to bottom showed that, as the stimulus level decreased, the amplitude of the peaks decreased and the latency increased. ABR wave amplitude was lower at 32 kHz than at 8 kHz in both young (Figure 1A, 1B) and geriatric (Figure 1C, 1D) marmosets. Aging did not affect ABR at 8 kHz, but the ABR waveform was not reliable at 32 kHz in the 10-year-old marmoset (Figure 1B, 1D).

ABR component II was elicited firstly and distinctly in common marmoset [28], so the ABR threshold was defined as the lowest intensity of each specified stimulus that generated an observable wave II. Comparisons of the ABR thresholds of all monkeys at stimuli of 2, 4, 8, 16, 22 and 32 kHz showed that ABR thresholds were not correlated with age at low frequencies (Figure 1E and Supplementary Figure 1), but there were positively correlations with age at 16 kHz (F=4.326, R^2^=0.1129, p=0.0451) and 32 kHz (F=15.80, R^2^=0.3172, p=0.0003; Figure 1F). A regression model revealed a rate of increase of 4.1 dB per year for ABR thresholds at 32 kHz, indicating that ABR thresholds increased with age at ultra-high frequencies in common marmosets, unlike age-related ABR thresholds increasing from 0.5-16 kHz in rhesus monkeys [37].

To analyze the associations of ABR thresholds with age individually, the 38 marmosets were divided into three groups, young (2-4 years of age, 20 animals), middle-aged (5-7 years of age, 11 animals), and geriatric (8-10 years of age, 7 animals) marmosets. Significant differences among these three groups were not observed at thresholds of 2-8 kHz, except that the young and geriatric groups differed at 2 kHz (Figure 1E). Age-related hearing loss was first observed at 16 kHz, at which the ABR threshold was 18 dB (p<0.01) higher in geriatric than in middle-aged marmosets, indicating that hearing sensitivity decreased with age. At 22 kHz, geriatric marmosets exhibited ABR thresholds 17 dB higher than in young animals (p<0.005) and 21 dB higher than in middle-aged animals (p<0.001). ABR thresholds at 32 kHz were 26 dB higher in geriatric than in young animals and 16 dB higher in geriatric than in middle-aged animals.

### Decreased age-related ABR amplitude at ultra-high frequency

To compare ABR peaks at a suprathreshold sound level in the three groups of monkeys, wave II was analyzed at 80 dB SPL across all of the stimuli from 2 to 32 kHz. The value of 80 dB SPL was chosen because ABR waveforms were distinct in most of monkeys studied, and peak II was chosen to analyze age-related changes because geriatric monkeys required higher stimulus levels than the other age groups to elicit peaks II and IV [37].

**Figure 2:**
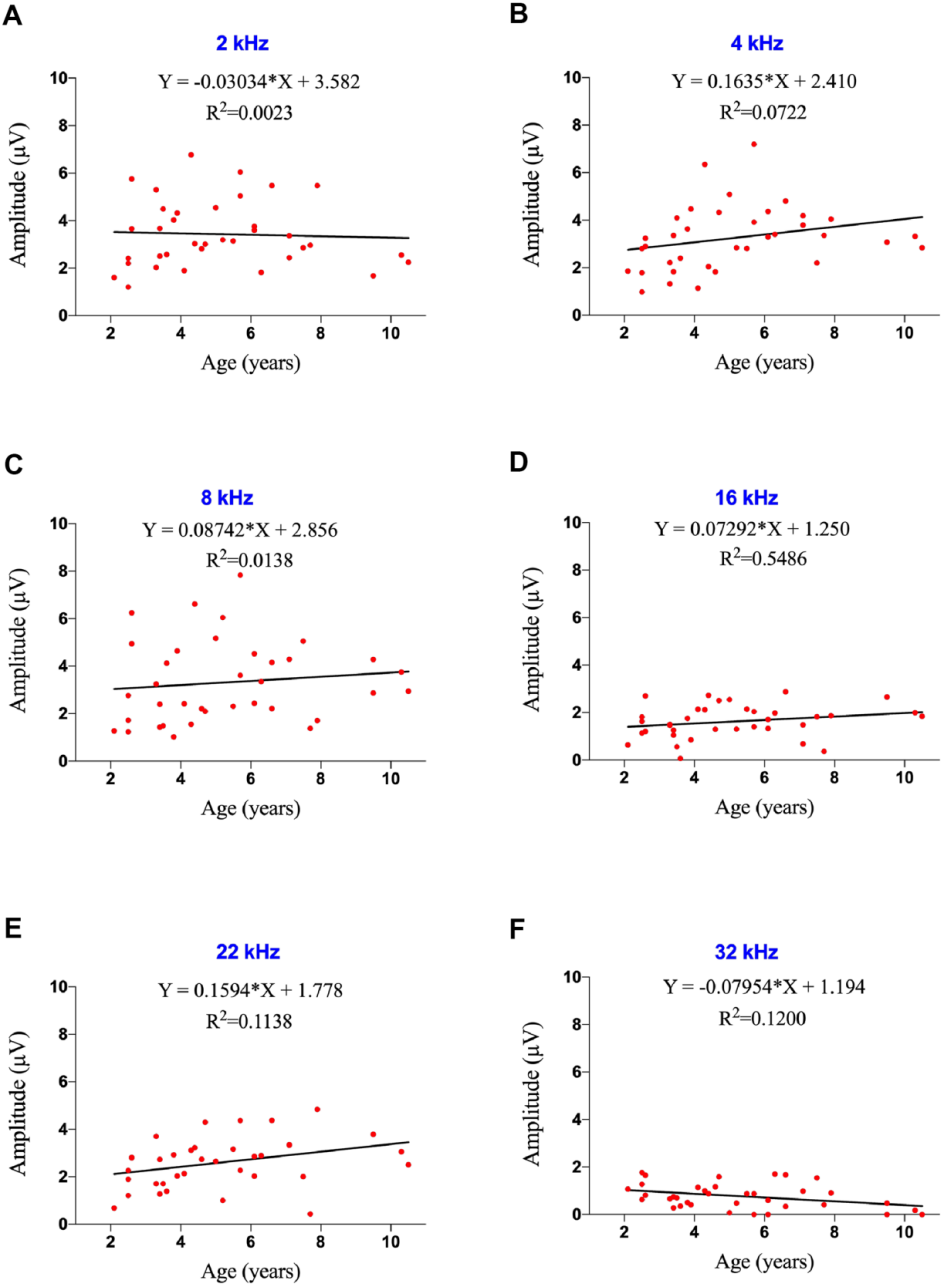
**Effects of age on ABR amplitude at ultra-high frequency in marmosets.** (**A**–**F**) Scatter plots and corresponding regression lines and regression equations for the relationships between ABR amplitudes (μV) of 36 individual marmosets and age (years) at frequencies of 2 kHz (**A**; R-squared linear=0.0023, P=0.7789); 4 kHz (**B**; R-squared linear=0.0722, P=0.1129); 8 kHz (**C**; R-squared linear=0.0138, P=0.4944); 16 kHz (**D**; R-squared linear=0.05486, P=0.1691); 22 kHz (**E**; R-squared linear=0.1138, P=0.0442); and 32 kHz (**F**; R-squared linear=0.1200, P=0.0385).

Age was calculated to one decimal place in years, and the correlation between age and ABR amplitude was analyzed. Regression equations showed that the ABR amplitude did not change with age at 2-22 kHz, with R^2^ values ranging between 0.0023 and 0.0722 (P>0.05 each; Figure 2A–2E). Age-related reductions in ABR amplitude were observed at 32 kHz (F=4.638, R^2^=0.1200, p=0.0385), with the amplitudes decreasing 0.07954 μV per year (Figure 2F). Peak amplitudes were lower at ultra-high frequency (32 kHz) than at low and middle frequencies (Figure 2).

The latencies of ABR component II were analyzed at all frequencies following stimulation at 80 dB SPL. However, there was no age-related difference in latency among these groups of marmosets (Supplementary Figure 2).

### DPOAE threshold and amplitude did not correlate with age

DPOAEs are responses generated when the cochlea is stimulated simultaneously by two pure tone frequencies, which are similar in rhesus monkeys and humans [38–40]. To our knowledge, only two previous studies assessed DPOAEs in marmosets, finding that DPOAEs tended to be slightly stronger in the right than in the left ear [41, 42]. Neither of these studies, however, analyzed the associations between DPOAE and age in marmosets.

**Figure 3:**
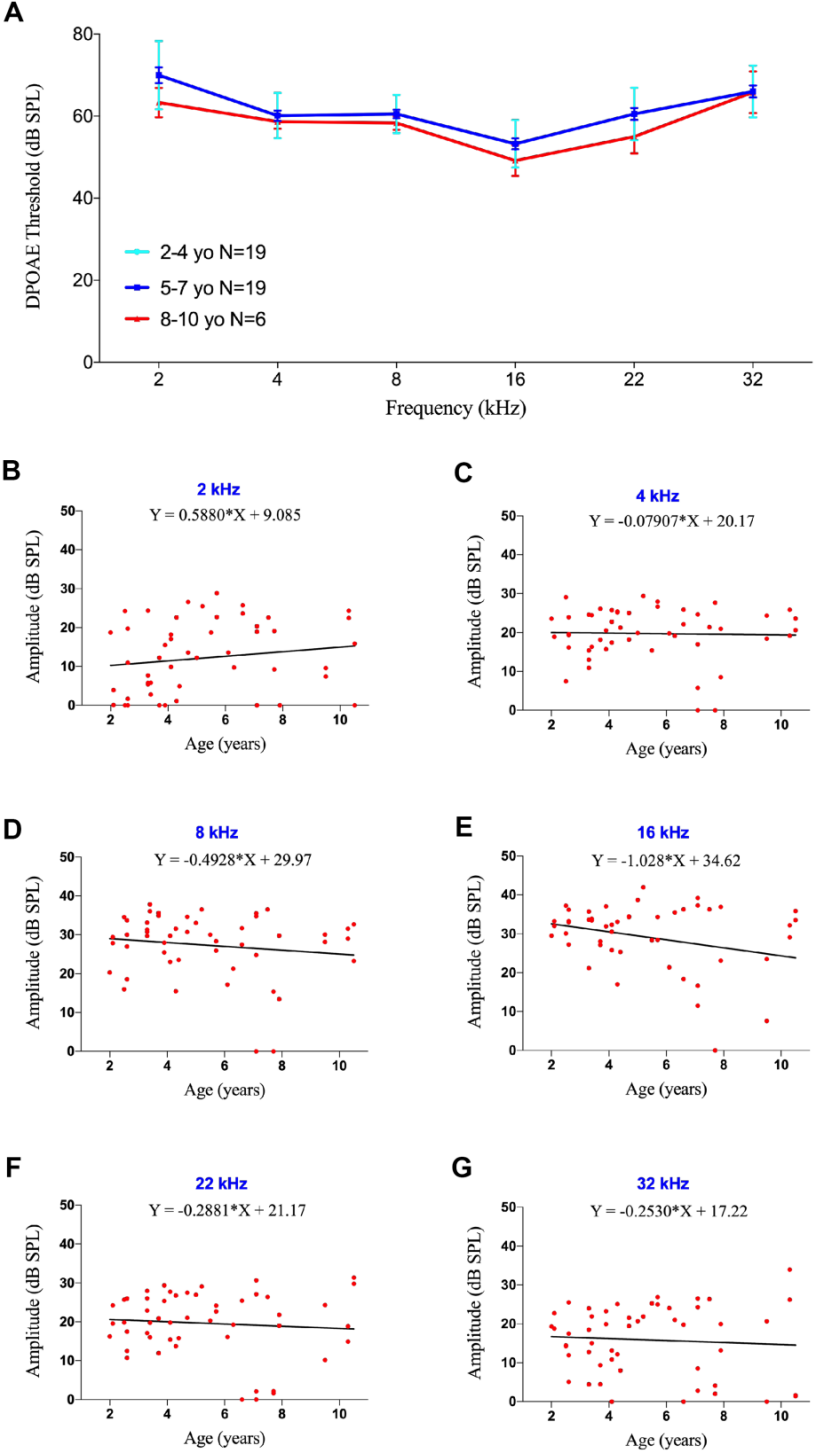
**DPOAE threshold and amplitude were not affected by age.** (**A**) Association between age and auditory thresholds determined by DPOAE. Data are presented as mean ± SEM. No significant differences were observed. (**B**–**G**) Scatter plots and corresponding regression lines and regression equations for the relationship between the DPOAE amplitudes (dB SPL) of 51 ears in 36 individual marmosets and age (years) at tone burst stimuli of 2 kHz (**B**; R-squared linear=0.02408, P=0.2769); 4 kHz (**C**; R-squared linear=0.00085, P=0.8391); 8 kHz (**D**; R-squared linear=0.02160, P=0.3035); 16 kHz (**E**; R-squared linear=0.07479, P=0.0522); 22 kHz (**F**; R-squared linear=0.00754, P=0.5446); and 32 kHz (**G**; R-squared linear=0.00488, P=0.6263).

DPOAEs were detected in 44 ears of marmosets at different frequencies (2-32 kHz), with 6 animals showing the occurrence of DPOAEs in both ears. The threshold was defined as the last detectable amplitude following auditory stimulation. Averaged DPOAEs did not vary distinctly at all frequencies, ranging from 53.3-66.1 dB SPL Figure 3A), although DPOAE thresholds were determined to be 40 dB SPL in several individual animals. Unlike ABR, DPOAE thresholds at all frequencies tested did not correlate with age (Supplementary Figure 3, p>0.05 each).

When the tested animals were divided into three age groups, as described in the ABR test, there were no significant difference in DPOAE thresholds among these three groups (Figure 3A), indicating that OHC function did not worsen with age in marmosets. Analysis of DPOAE amplitudes at different frequencies also did not differ by age (Figure 3B–3G, p>0.05 each).

### Auditory periphery neural degeneration in geriatric common marmosets

**Figure 4:**
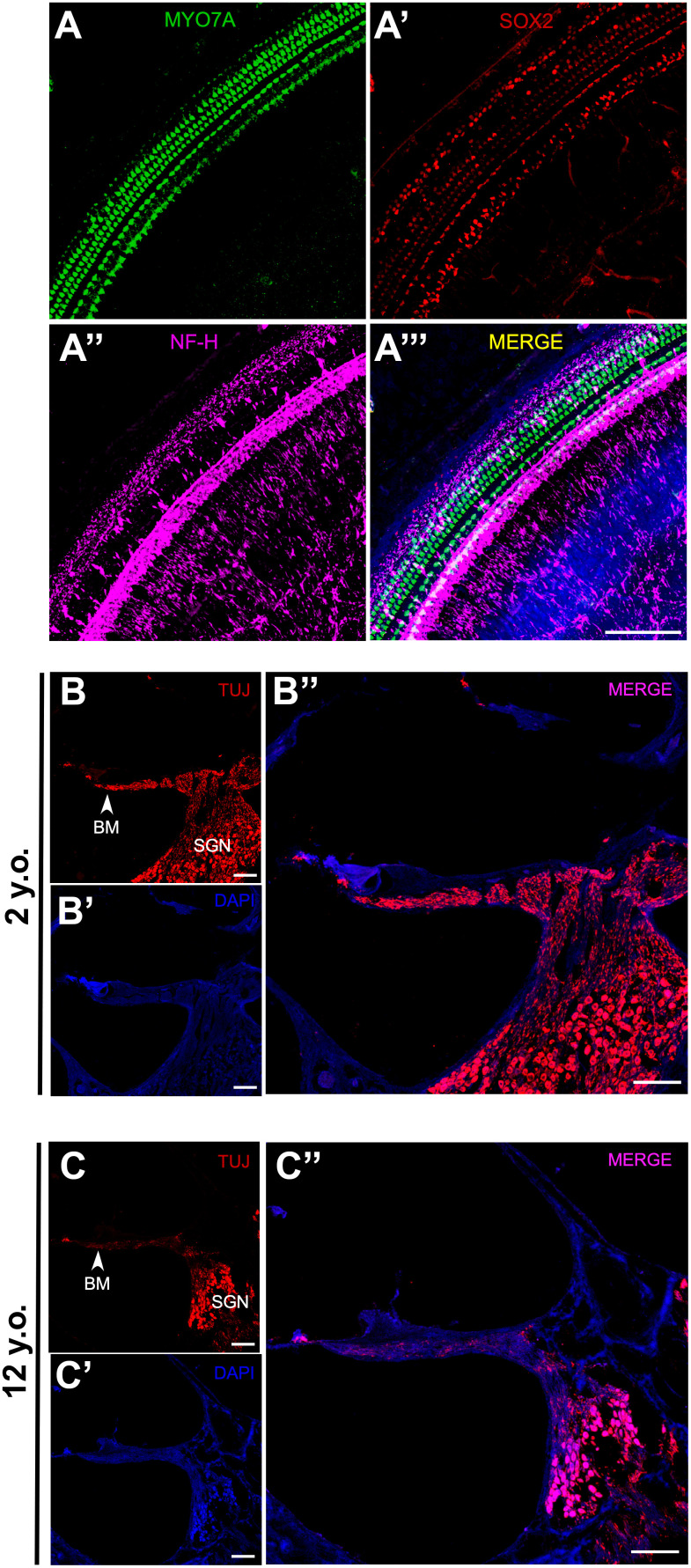
**Effects of age on SGN density in marmosets.** (**A**–**A”**) Confocal whole-mount immunofluorescence images of the middle turn of a cochlea in a 2-year-old marmoset. (**A**) Hair cells labeled with MYO7A. (**A’**) Supporting cells positive for SOX2. (**A”**) Neural fibers labeled with NF-H. (**A”’**) Merged channels figure, MYO7A (green), SOX2 (red), NF-H (magenta), DAPI (blue). (**B**–**B”**) Confocal images of a cochlear cryosection from a 2-year-old marmoset. SGNs labeled with TUJ. TUJ-positive neurofilaments connect the basilar membrane (BM) to the modiolus. (**C**–**C”**) Confocal images of a cochlear cryosection from a 12-year-old marmoset. Scale bar: 100 μm.

To explore the mechanism of age-related ABR changes in common marmosets, the cochleae were extracted from the temporal bone of a 2-year-old marmoset. Whole mount preparations of the cochlea revealed hair cells in the organ of Corti. Hair cells (HCs), supporting cells and neural connections in the cochlea were labeled immunohistologically with antibodies to MYO7A, SOX2 and NF-H, respectively (Figure 4A–4A’’’). This methodology, however, could not show all putative myelinated spiral ganglion cell bodies. To assess SGN associated neural presbycusis, the morphological features of basal-modiolus semi-thin sections from a 2- year-old and a 12-year-old marmoset were compared.

**Figure 5:**
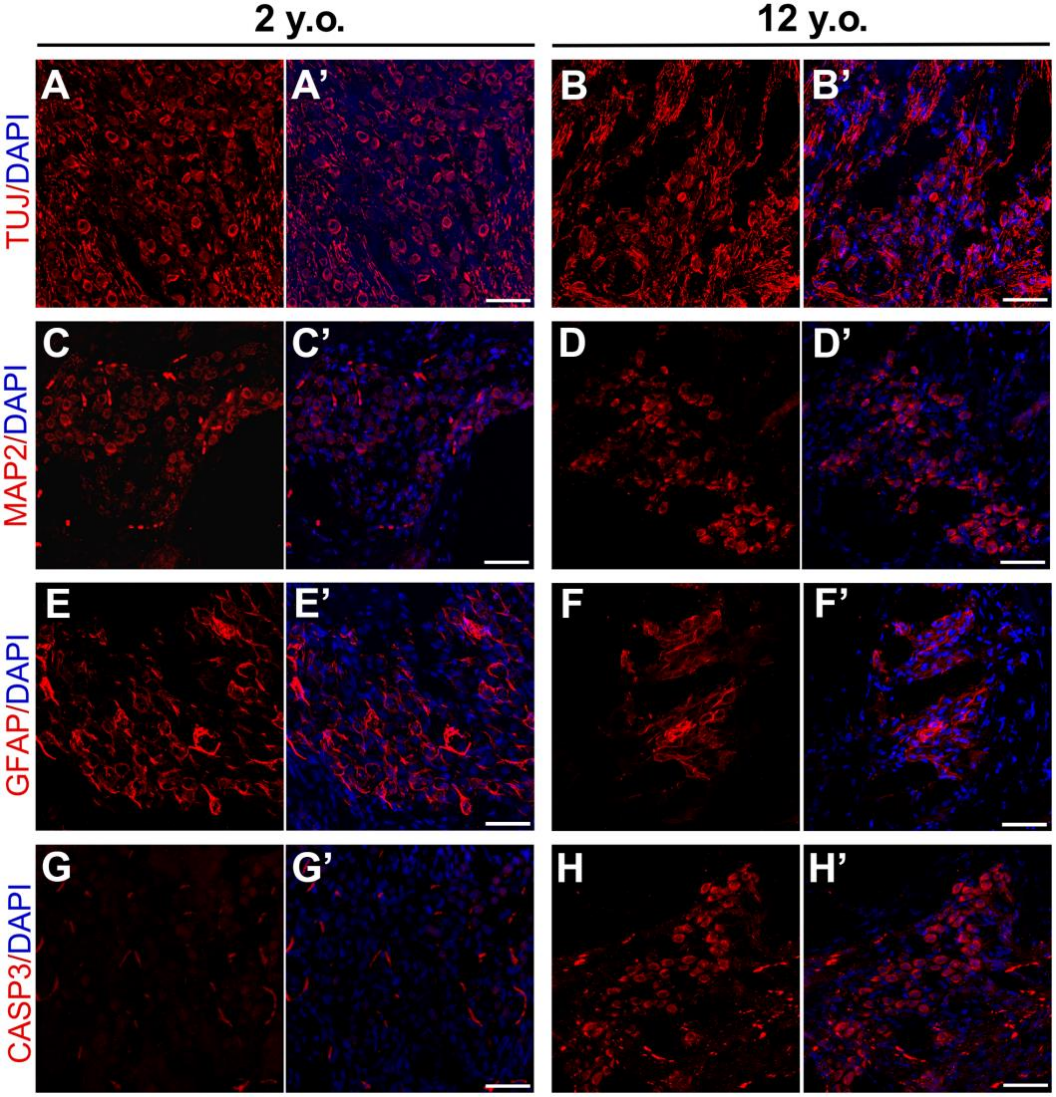
**Effects of age on expression patterns of neuronal and glial markers in marmosets.** Confocal immunofluorescence images of cryosections of the modiolus regions of 2-year-old and 12-year-old marmosets. SGNs labeled with TUJ in the (**A**, **A**’) 2-year-old and (**B**, **B’**) 12-year-old marmosets. MAP2 positive neurons in the modiolus of the (**C**, **C’**) 2-year-old and (**D**, **D’**) 12-year-old marmosets. GFAP2 positive glia were in the (**E**, **E’**) 2-year-old and (**F**, **F’**) 12-year-old marmosets. Apoptosis was labelled by cleaved Caspase-3 in 2-year-old marmoset (**G**, **G’**) and 12-year-old marmoset (**H**, **H’**). Scale bar: 50 μm.

The basilar membrane (BM) morphology did not differ in the inner ears of these two marmosets (Figure 4B, 4C). TUJ1 antibody, which reacts with beta-tubulin III, was found to stain SGN in developing marmosets [30]. The packing density of SGNs in the modiolus was much higher in the 2-year-old than in the 12-year-old marmoset (Figure 4B, 4C). Several non-cell cavities were observed in the modiolus of the older marmoset, indicating that the degeneration of SGNs leads to age-related hearing loss, rather than HC loss. In addition to the reduction of TUJ1-positive cells in the cochlea of the geriatric marmoset, the remaining TUJ1-positive cells were both disorganized and swollen in this animal (Figure 5A, 5B).

To better assess modiolus pathology in marmosets, thin sections from the cochlea of a geriatric marmoset were stained with antibodies to additional cell types. In a previous study in rodents, microtubule associated protein 2 (MAP2) was tightly regulated in rodent SGNs undergoing repair, suggesting that MAP2 positive cells may be involved in the regeneration of primary auditory neurons after injury [43]. Many MAP2 positive cells were observed in the cochleae of both the young and geriatric marmosets, with no distinct difference between them (Figure 5C, 5D), indicating that primates have limited SGN regeneration capacity after birth. Age-related changes in the expression of glial fibrillary acidic protein (GFAP) were observed in rodent cochlear nuclei [44], suggesting that the increase in the number of GFAP positive astrocytes may be due to alterations in auditory nerve fibers and trophic interactions with post-synaptic cells. The current study found that many GFAP positive cells were around SGNs in the young marmosets (Figure 5E), but that the number of GFAP positive cells in the modiolus was lower in the geriatric marmosets (Figure 5F). This decrease in GFAP positive cells reflected astrocyte degeneration in the cochlea, accompanying neuron degeneration.

To validate neural degeneration, we labelled cleaved caspase-3 in young and old marmoset. Caspase-3 is defined as the effector caspase that cleave key regulatory or structural proteins and in particular activate apoptotic nucleases [45] and was used to detect apoptosis SGN of rodents after ototoxicity [46, 47]. We observed plenty of cleaved caspase-3 positive cells in the modiolus of geriatric marmoset cochlea (Figure 5G), and rare cleaved caspase-3 positive cells in the young one (Figure 5H). The arising of caspase-3 positive cells in modiolus of aged marmoset indicated SGN apoptosis appeared during aging, which demonstrated neural apoptosis was the main reason for ARHL.

### Auditory dys-synchrony at ultra-high frequency in geriatric rhesus monkeys

To explore auditory function at ultra-high frequencies in another NHP, DPOAE and ABR were tested in 40 rhesus monkeys aged 2 to 24 years. Rhesus monkeys age approximately three times faster than humans, making these animals roughly equivalent to humans aged 6 to 72 years. ABR results did not differ significantly in male and female rhesus monkeys (data not shown).

**Figure 6:**
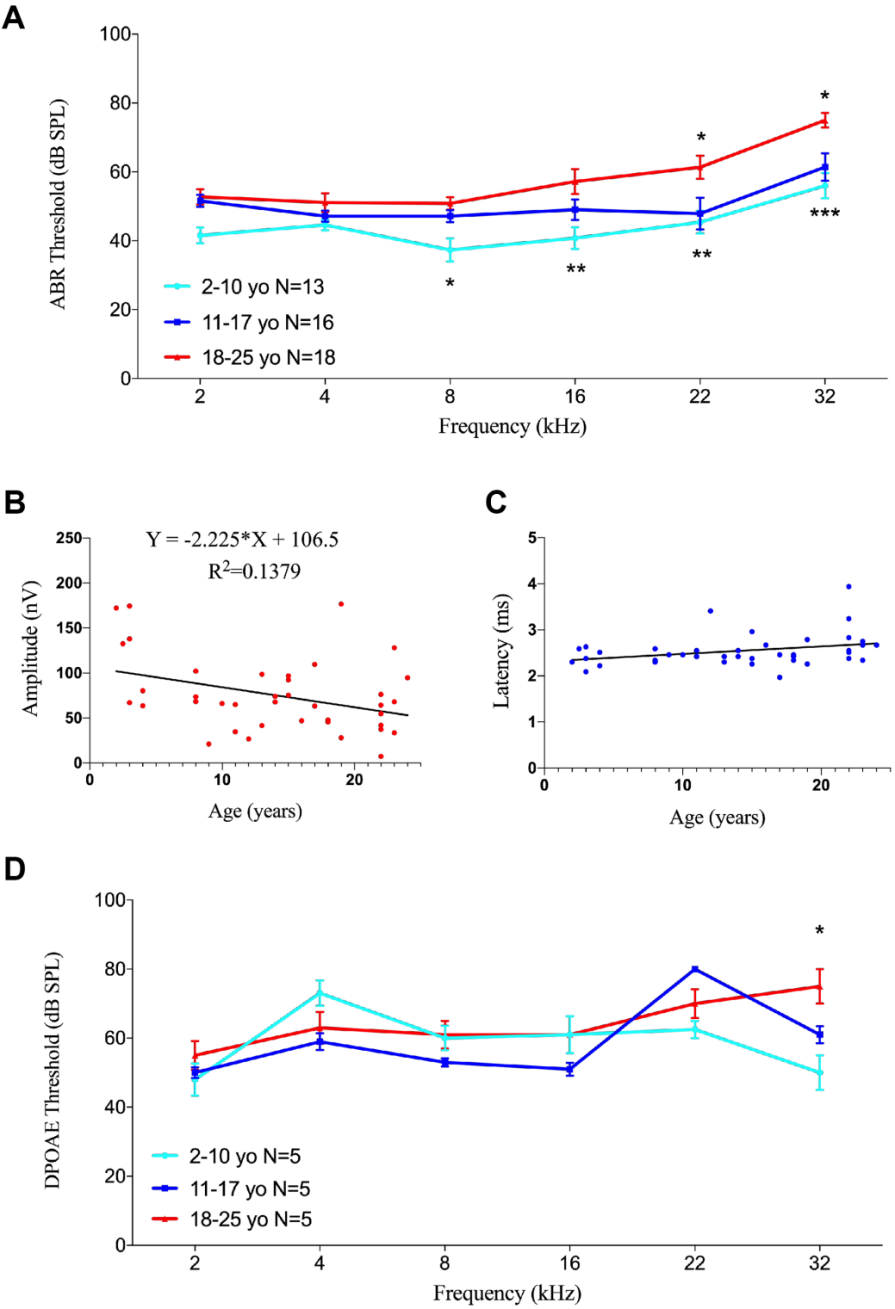
**Effects of age on auditory function in rhesus monkeys.** (**A**) Age-related changes in auditory thresholds determined by ABR. Data are presented as mean ± SEM. Significant differences in ABR threshold between the groups are marked. Compared to 2-10 year-old animals, ABR thresholds increased in aging rhesus monkeys from 8-32 kHz. ABR thresholds increased at 22 and 32 kHz compared aging monkeys with 11-17 y.o. ones. *P < 0.05; **P < 0.01; ***P < 0.001 by two-way ANOVA for comparisons of ABR thresholds in 2-10-year-old and 11-17-year-old rhesus monkeys. (**B**) Scatter plot and corresponding regression line and regression equation for the relationship between ABR amplitudes (nV) and ages (years) of 40 individual rhesus monkeys at a frequency of tone burst stimuli of 22 kHz (R-squared linear= 0.1379, P=0.0183). (**C**) Scatter plot and corresponding regression line and regression equation for the relationship between ABR latencies (ms) and ages (years) of the 40 rhesus monkeys at a frequency of 22 kHz (R-squared linear= 0.1010, P=0.0456). (**D**) Association between age and auditory thresholds determined by DPOAE in rhesus monkeys. Data are presented as mean ± SEM. *P < 0.05 by two-way ANOVA for comparisons of DPOAE thresholds in 2-10-year-old and 18-25-year-old animals.

The 40 animals were divided into three groups: young (2-10 years of age, 13 animals), middle-aged (11-17 years of age, 16 animals), and geriatric (18-25 years of age, 18 animals). Auditory functions at 8 to 32 kHz were significantly poorer in geriatric than in young monkeys (Figure 6A). Compared with young rhesus monkeys, ABR thresholds in geriatric monkeys were 13.5, 16.5, 16.0 and 19.0 dB higher at 8 (p<0.05), 16 (p<0.01), 22 (p<0.01) and 32 (p<0.005) kHz, respectively. ABR thresholds also differed between middle-aged and geriatric monkeys, but only at ultra-high frequencies of 22 and 32 kHz (p<0.05 each), indicating that the rising ABR threshold at high frequency was incipient audiogram indicators of ARHL.

Because ABR wave II was distinct in rhesus monkeys [37], its amplitudes and latencies were analyzed to evaluate auditory function. Linear regression analysis showed an age-related decrease in ABR amplitude of 70 dB at 22 kHz (F=6.080, R^2^=0.1379, p=0.0183), with a slope of -2.225 relative to age (Figure 6B). In contrast, the linear regression equation of latency under the same conditions showed a significant prolongation (F=4.271, R^2^=0.1010, p=0.0456), with a slope of 0.01627 (Figure 6C).

Testing of DPOAE in 15 rhesus monkeys, five from each group, showed no age-related differences in DPOAE thresholds, except at 32 kHz. The DPOAE threshold was 25.0 dB higher in geriatric than in young rhesus monkeys at 32 kHz (p<0.05), but there were no significant differences at other frequencies (Figure 6D), indicating that DPOAE threshold was stable with age in rhesus monkeys.

## DISCUSSION

The present study utilized audiometry and pathology to assess neural presbycusis at ultra-high frequency in aged marmosets and rhesus monkeys. Neural degeneration in aged common marmosets indicated neural degeneration that contributed to presbycusis in NHPs.

Presbycusis manifests as a progressive reduction in high frequency hearing sensitivity over an increasing range of frequencies, eventually affecting the frequency range relevant for speech. NHPs are ideal animal models to identify specific mechanisms and physiological deficits underlying presbycusis, and to develop strategies for its treatment in human. NHPs are less frequently used than humans and rodents in studies of ARHL, because NHPs are relatively rare and are expensive to purchase and maintain. Humans and NHPs have strong similarities in genetics and in the anatomy and neurophysiology of the auditory nervous system, suggesting that analyses in NHPs could enhance understanding of ARHL in humans [37]. ARHL can be assessed by hearing tests and histologic examination. Our results showed that ABR and DPOAE in marmosets and rhesus monkeys responded similarly to variations in sound frequencies, indicating that New World and Old World monkeys have similar age-related alterations in audiograms. By establishing hearing baselines for these two NHPs at different ages, we were able to analyze the mechanism of presbycusis in both species. The present study included 38 marmosets and 40 rhesus monkeys of different ages from isolated inbred colonies, excluding the effects of other influences, such as genetic and environmental factors. Studies in aging NHPs may help determine the mechanism of development of ARHL and methods to intervene in its development.

Marmosets and rhesus monkeys have been widely used to assess the effects of aging on hearing [48, 49], with previous studies in these species showing age-associated increases in audiometric thresholds across the frequency spectrum [23, 28, 37, 50]. Similar to these studies, we found that ABR thresholds were poorer in older than in younger animals, indicating that auditory function declines with age [23, 28, 37, 50]. Loss of threshold sensitivity in the high frequency region of the hearing spectrum is an early sign of presbycusis, with the earliest effects of age observed at the highest test frequencies (8 kHz) [2]. Although age-related changes in auditory BAEP thresholds at high frequencies have been reported, BAEP thresholds were not correlated with age in marmosets, perhaps due to the small number of animals [28]. To our knowledge, the present study is the first to show reductions in ABR sensitivity at high frequencies (22 and 32 kHz) in geriatric marmosets, with changes in both amplitude and latency. ABR thresholds in rhesus monkeys were previously tested at frequencies ≤16 kHz, but age-related changes in ABR thresholds were not evaluated [23, 27, 37, 50, 51]. The present study assessed auditory function at frequencies from 2 to 32 kHz, finding that ABR thresholds at 16 and 32 kHz were significantly poorer in geriatric than in younger NHPs. Age-associated changes in ABR thresholds at high frequencies are indicators of ARHL. A study of two 24-year-old and two 9-year-old rhesus monkeys showed that auditory sensitivity at 32 Hz was lower in one of the 24-year-old than in one of the 9-year old animals [26], which is consistent with our results. ABR thresholds at high frequency (>16 kHz) appear to be sensitive indicators of early ARHL in NHPs.

ABR and DPOAE have rarely been detected simultaneously in geriatric NHPs, and there have been few descriptions of age-associated changes in inner ear histology in NHPs. The results of this study indicated that two species of NHPs experienced progressive high frequency hearing impairment, which increased in severity with natural aging. DPOAE, which has been rarely tested in monkeys [38, 39, 50], was stable with age, indicating that OHCs did not deteriorate in geriatric NHPs. Because the microphone output of 20 dB was not amplified, our DPOAE thresholds were higher than previously reported [42]. Histologic results also showed that neural degeneration occurred prior to HC degeneration in NHPs. The reliability of DPOAE measurements in marmosets in the present work was similar to that reported in a previous study, with subtle variations observed in animals of similar age [41, 42]. The present study found that DPOAE in marmosets and rhesus monkeys was unaffected by age, demonstrating that OHC function was not altered by aging. Similar to humans, aged NHPs showed reduced ABR with normal DPOAE, or dys-synchrony [2].

The results of hearing tests showed that ARHL in NHPs could be characterized as sensorineural hearing loss. Analysis of ABR waves and cochlear histology may reveal the mechanism underlying the development of ARHL. Waves II and III are generated by the cochlear nucleus, superior olivary nucleus and lateral lemniscus [37], with age-related alterations in wave amplitude and latency indicating that lesions were located in the cochlear nucleus or SGN. As there was no ABR wave in aging monkey, so it was hard to distinguish if central auditory system was also dysfunction. Based on our data, ultra-high frequency auditory dysfunction was caused by neural degeneration, rather than HCs. Assessment of cochlear pathology in geriatric monkeys showed degeneration of SGNs, indicating that neural degeneration occurred early in the development of ARHL and that preservation of SGNs may contribute to the treatment of ARHL.

Although age-related hearing loss at high frequencies was previously reported in rhesus monkeys, no systematical acoustic study had assessed ABR and DPOAE in marmosets. Our assays, in 38 marmosets, showed that ABR thresholds, amplitudes and latencies at high frequencies were poorer in geriatric than in younger marmosets. Alterations in ABR thresholds at ultra-high frequencies (22-32 kHz) were observed in marmosets aged 5-7 years, with changes at lower frequency (16 kHz) observed in marmosets aged 8-10 years, indicating that ARHL started at high frequencies and subsequently was extended to lower frequencies. Evaluation of ultra-high frequency hearing was not practical in marmosets, as their primary vocalizations were at frequencies <8 kHz [52]. Ultra-high frequency ABR alterations were also observed in geriatric rhesus monkeys, indicating that reduced acoustic function at ultra-high frequencies was common to NHPs. Tests of ABR at ultra-high frequencies in aging humans may contribute to a diagnosis of presbycusis, although ABR is rarely tested at frequencies >20 kHz.

Wave I is generated by cranial nerves, and wave II by cochlear nuclei [53]. Because age-associated changes in wave II latency of BEAP were observed in marmosets, this study compared wave II amplitudes and latencies at different ages [28]. Age-related changes in wave II indicated that neural degeneration was the primary cause of presbycusis in primates, although determining the involved parts of the auditory nervous pathway requires further study.

Unlike the age-related changes in ABR, DPOAE at high frequency was stable in geriatric marmosets and rhesus monkeys. Age-related reductions in DPOAE have been reported to be at frequencies <10 kHz [27, 39]. However, we did not observe OHC dysfunction at high frequencies in geriatric NHPs. ARHL in NHPs was not caused by OHC degeneration, and SGN degeneration was detected in geriatric marmosets. Consistent with results in rhesus monkeys, SGNs showed degeneration in marmosets [23]. SGN degeneration was more distinct in geriatric marmosets compared to rodents, while was similar to human cochleae [54].

Although neural degeneration may be the main cause of ARHL, further studies are needed to validate these findings. For example, cochleae from larger number of marmosets and/or rhesus monkeys should be assessed histologically. Because synapses are vulnerable to degeneration, we attempted to stain cochlear synapses in marmosets with CTBP2. However, because we used tissue samples from animals that died naturally, it was difficult to harvest tissue immediately after death. Mitochondria analysis could be performed in aging monkeys in the future, as mitochondria dysfunction was proved in aging rodents [55, 56].

NHPs may be better animal models than rodents for research on ARHL. Further studies of audiograms and histology are needed to evaluate the mechanism of development of ARHL. Our study, showing neural presbycusis in two species of NHP, indicate that neural presbycusis may be a universal characteristic of ARHL.

## MATERIALS AND METHODS

### Subjects

Thirty-eight common marmosets *(Callithrix jacchus)* were obtained from the Institute of Neuroscience, the Key Laboratory of Primate Neurobiology, CAS Center for Excellence in Brain Science and Intelligence Technology, Chinese Academy of Sciences. The animals were aged 1 to 11 years, with an average life span in a breeding colony of around 10-12 years [57]. None of the animals in this study had behavioral disorders and all had clear ear canals and intact tympanic membranes, as confirmed by otoscopic examination. No animal had a history of loud noise exposure, ear trauma, or ototoxic drug treatment. All experimental procedures adhered to the guidelines of the Animal Advisory Committee at the Institute of Neuroscience, Key Laboratory of Primate Neurobiology, CAS Center for Excellence in Brain Science and Intelligence Technology, Chinese Academy of Sciences.

Forty rhesus monkeys aged 2 to 24 years were detected from Sichuan Primed Shines Bio-tech Co., Ltd. All experimental procedures adhered to the guidelines of the company and of the above Animal Advisory Committee and complied with laboratory animal ethics.

### Anesthesia

Before anesthesia, the animals were fasted for more than 4 hours to prevent reflux from obstructing the airways. Marmosets were injected intramuscularly on the left hind leg with 0.12 mL atropine 10 minutes before inhalation anesthesia, to prevent gastroesophageal reflux. The animals were intramuscularly injected on the right hind leg with 0.1 mL ketamine. A custom-made mask was applied for oxygen and isoflurane inhalation to maintain good anesthesia. Rhesus monkeys were intramuscularly injected with 50 mg/mL ketamine to yield a dose per animal of 10 mg/kg.

During the anesthesia process, animals were treated with ECG and their blood oxygen was monitored to ensure their safety. Upon completion of testing, animals were kept warm and monitored until the effects of anesthesia disappeared, and were then returned to their home cages.

### ABR

All recordings were performed in an electrically shielded and sound-attenuated chamber. ABR recordings were performed using 12 mm long, 27 gauge needle electrodes. Before insertion, the skin was carefully cleaned with alcohol. The recording electrode (+) was inserted subcutaneously at the intersection of the apex line of the two ears and the midline of the skull, the reference electrode (-) was inserted at the dorsal base on the stimulated side, and the ground electrode was subcutaneously inserted into the veutro part of the tail. Electrode impedance was measured after they were inserted into the skin, with impedance being below 1 kΩ for all recordings.

Auditory stimuli, consisting of tone bursts of duration 1.0 ms with 0.1 ms rise and fall times, were generated by an acoustic stimulator (RZ6, Tucker-Davis Technologies, Inc.) to induce estimated ABR peaks. These signals were delivered to a speaker (MF1 Multi-Function Speaker, Tucker-Davis Technologies, Inc.) inserted into the animal's ear and attached with a 5 cm-long tube (Tucker-Davis Technologies). Stimulus protocols for tone bursts were programmed using SigGenRZ software (Tucker-Davis Technologies, Inc.). The bioelectrical ABR signals recorded from the subdermal electrodes were transferred to a head stage (RA4LI, Tucker-Davis Technologies, Inc.) and forwarded to a preamplifier (RA4PA, Tucker-Davis Technologies, Inc.) with 20-fold amplification.

The responses to an average of 512 stimuli were amplified 100,000 times and filtered with high and low bandpasses of 100 Hz and 3 kHz, respectively. The stimulus output was reduced in 5 dB steps until the response was undetectable, with the first stimulus intensity that was unable to evoke any response defined as the threshold. Generally, wave II outputs became visible as stimulus intensities decreased. Tone bursts were fixed at 20 Hz. The effects of stimulus frequencies of 2 to 32 kHz on ABR latencies and amplitudes were evaluated at a stimulus intensity of 80 dB SPL. The latencies and amplitudes of the peaks were measured to the nearest 0.01 ms and the nearest 0.01 WV, respectively.

### DPOAE

A probe microphone/loudspeaker system with two loudspeaker ports (ER10B+, Etymotic Research, Inc.) was inserted into the ear canal, and two loudspeakers (MF-1, Tucker-Davis Technologies, Inc.) were connected to the ER10B+ ports. Acoustic stimuli were delivered from an analog input/output (I/O) device (RZ6, Tucker-Davis Technologies, Inc.), through an electrostatic speaker driver (ED-1, Tucker-Davis Technologies, Inc.), to the MF-1 speakers. Each primary tone was delivered through a separate loudspeaker to avoid the generation of intermodulatory sounds, with responses recorded in an electrically shielded and sound-attenuated chamber. No spurious intermodulation products generated by the electronics or by the acoustics of the canal were detectable at the DPOAE frequencies assessed in these recordings. The data-acquisition module was connected to a personal computer (WS-8 workstation, Tucker-Davis Technologies, Inc.).

The sound stimulus consisted of simultaneous permanent pure tones at two different frequencies (f2/f1 ratio = 1.22), decreasing from 80 dB to 10 dB (L1 = L2) in 5 dB steps. DPOAEs were measured at six frequencies: 2, 4, 8, 16, 22, and 32 kHz. The DPOAE signals were displayed using TDT software.

### Data analysis

Data were analyzed by analysis of variance (ANOVA), with statistical significance defined as P<0.05. Regression analyses were conducted hierarchically, with age (as a continuous variable) entered to account for between-subject variability, where R-squared equals the coefficient of determination.

For ABRs, the peak amplitude of each wave was defined as the average of the evoked potentials from the summit of the peak to the lowest points of the nearest troughs on the two sides. The peak latency for each peak was defined as the time from stimulus onset to the summit of the peak. Results of tests in which no observable waveform was present at any level for a tone stimulus were excluded from analysis.

For DPOAEs, the amplitude of each frequency at 80 dB SPL was defined as the numerical measure of sound level produced at a frequency of 2f1-f2 compared with background noise. Values below zero were scored as zero. The hearing threshold at each frequency was defined as the last detectable amplitude produced in response to the acoustic stimulation. Thresholds were determined as 80 dB SPL if DPOAE could not be elicited.

### Immunohistochemistry

For the marmosets that died a natural death during the study period, the temporal bone region was dissected immediately after death and fixed 24 hours with 4% paraformaldehyde in phosphate-buffered saline (PBS; pH 7.2). The tissue samples were transferred to 0.12 M EDTA for decalcification. EDTA was refreshed weekly for 2 weeks, and decalcified tissue was trimmed at each change of EDTA solution.

Decalcified cochleae were dissected into quarter turns, and the pieces were incubated in blocking reagent (8% normal donkey serum, 0.05% Triton X-100 in PBS) for 1 h at room temperature. The pieces were subsequently incubated overnight at room temperature with 1:300 anti-MYO7A primary antibody (Proteus Biosciences), 1:300 anti-TUJ1 primary antibody (BioLegend, 801202), 1:300 anti-NF-H primary antibody (Millipore, AB5539), 1:100 anti- Cleaved Caspase-3 (Cell Signaling Technology, 9661) or 1:300 anti-SOX2 primary antibody (R&D, AF2018). The tissue samples were subsequently washed three times with PBS and counterstained with 1:500 Alexa Fluor secondary antibodies (Invitrogen) for 1 hour, including donkey anti-rabbit Alexa Fluor 546 (A-21123), donkey anti-mouse Alexa Fluor 647 (A-31571) and donkey anti-rabbit Alexa Fluor 488 (A-21206). Nuclei were simultaneously stained with 5 mg/ml of a 1:1000 dilution of DAPI (D3571, Invitrogen). Confocal images were acquired on a Leica TCS SP8 laser confocal microscope using a 20× glycerin-immersion lens and the images were processed with ImageJ software.

### Cryo-embedding and sectioning of cochleae

Cochleae, fixed and decalcified as described above, were incubated in 30% sucrose dehydration solution for 24 h at room temperature. The cochleae were transferred into OCT (Sakura Finetek USA, CA90501) in parallel with the cochlear axis while avoiding air bubbles. The tissues were placed in a -80° C freezer overnight followed by final orientation and sectioning into 10 μm thick samples using a freezing microtome 400 (LEICA CM3050S).

The resultant slides were incubated in blocking reagent (8% normal donkey serum, 0.05% Triton X-100) for 1 h at room temperature. After washing, the sections were incubated overnight at room temperature with 1:300 dilutions of primary anti-TUJ1 antibody (Bio Legend, 801202), primary anti-NF-H antibody (Millipore, AB5539), primary anti-SOX2 antibody (R&D, AF2018), primary anti-MAP2 antibody (SYSY, 188011) and primary anti-GFAP antibody (Novus, NB300-141). The tissue samples were washed three times with PBS and incubated for 1 hour at room temperature with 1:500 dilutions of the Alexa Fluor conjugated secondary antibodies (Invitrogen), including donkey anti-rabbit Alexa Fluor 546 (A-21123), donkey anti-mouse Alexa Fluor 647 (A-31571) and donkey anti-rabbit Alexa Fluor 488 (A-21206). Nuclei were stained with 5 mg/ml of a 1:1000 dilution DAPI (D3571, Invitrogen). Confocal Images were acquired on a Leica TCS SP8 laser confocal microscope using 20× and 40× glycerin-immersion lenses, and all images were processed with ImageJ software.

## Supplementary Material

Supplementary Figures
