# Supplementary material for: Neural presbycusis at ultra-high frequency in aged common marmosets and rhesus monkeys
Source: Aging (Albany NY). 2021 Apr 27;13(9):12587–606. doi: 10.18632/aging.202936 (PMC8148503; doi:10.18632/aging.202936)
Supplement: Supplementary Figures [file aging-13-202936-s001.pdf]

SUPPLEMENTARY FIGURES

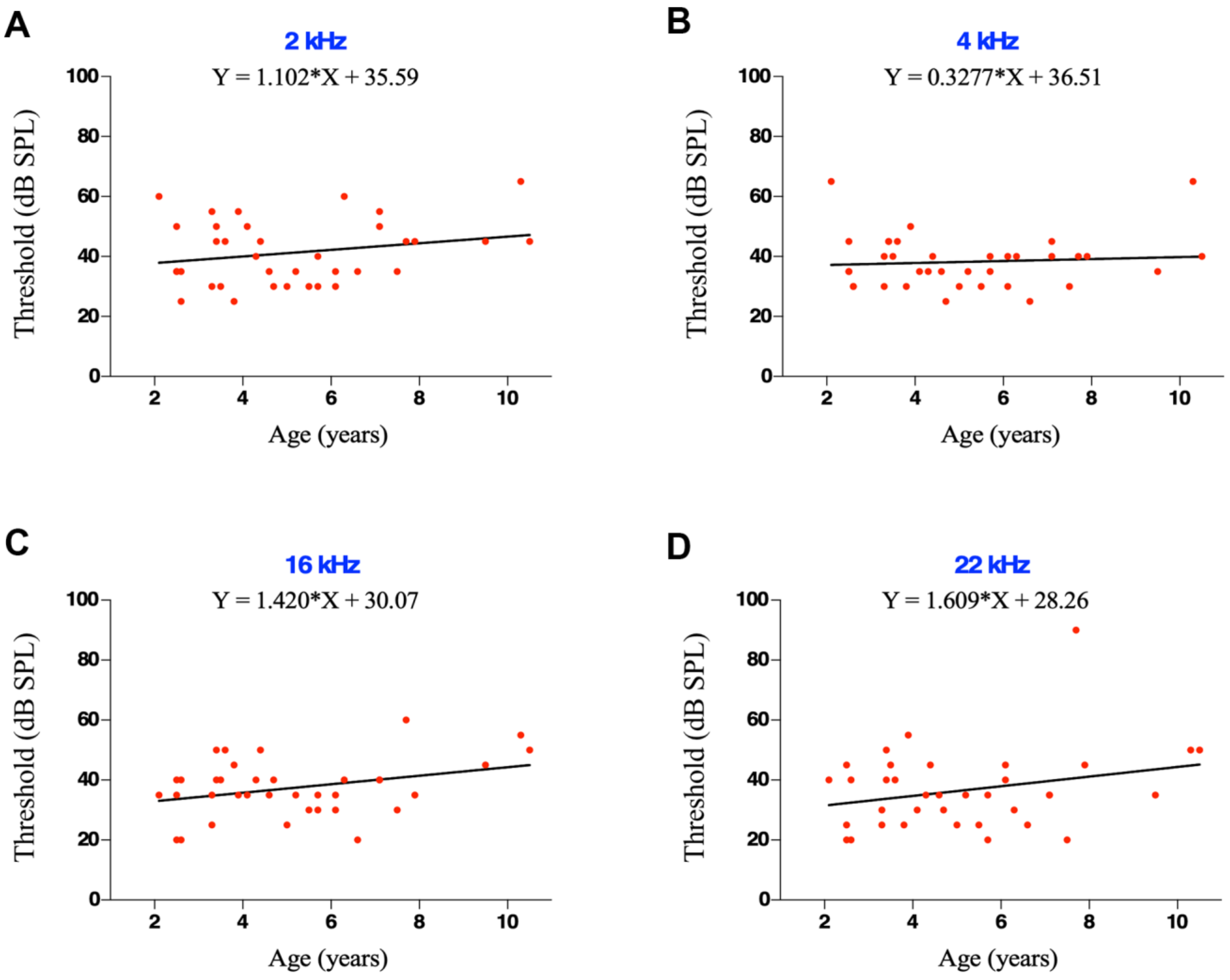

Supplementary Figure 1. Effects of age on ABR thresholds at low frequencies in marmosets. (**A**–**D**) Scatter plots and corresponding regression lines and regression equations for the relationships between ABR thresholds (dB SPL) of 36 individual marmosets and age (years) at frequencies of 2 kHz (**A**; R-squared linear=0.05357, P=0.1744); 4 kHz (**B**; R-squared linear=0.00669, P=0.6352); 16 kHz (**C**; R-squared linear=0.1129, P=0.0451); and 22 kHz (**D**; R-squared linear=0.07257, P=0.1121).

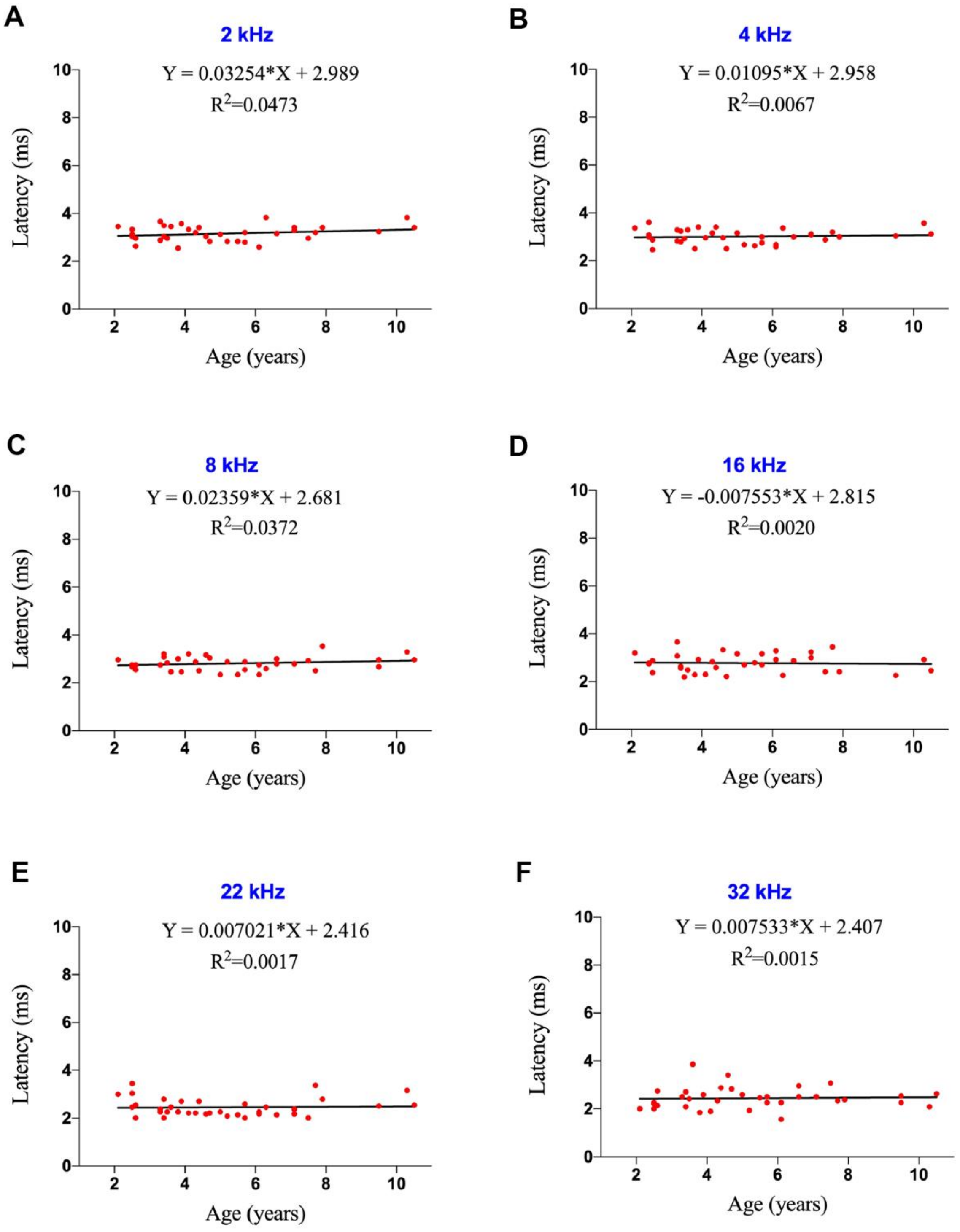

Supplementary Figure 2. Effects of age on ABR latencies in marmosets. (**A**–**F**) Scatter plots and corresponding regression lines and regression equations for the relationships between ABR latencies (ms) of 36 individual marmosets and age (years) at frequencies of 2 kHz (**A**; R-squared linear=0.04724, P=0.2024); 4 kHz (**B**; R-squared linear=0.00671, P=0.6349); 8 kHz (**C**; R-squared linear=0.03602, P=0.2676); 16 kHz (**D**; R-squared linear=0.00197, P=0.7971); 22 kHz (**E**; R-squared linear=0.00170, P=0.8113); and 32 kHz (**F**; R-squared linear=0.00052, P=0.8963).

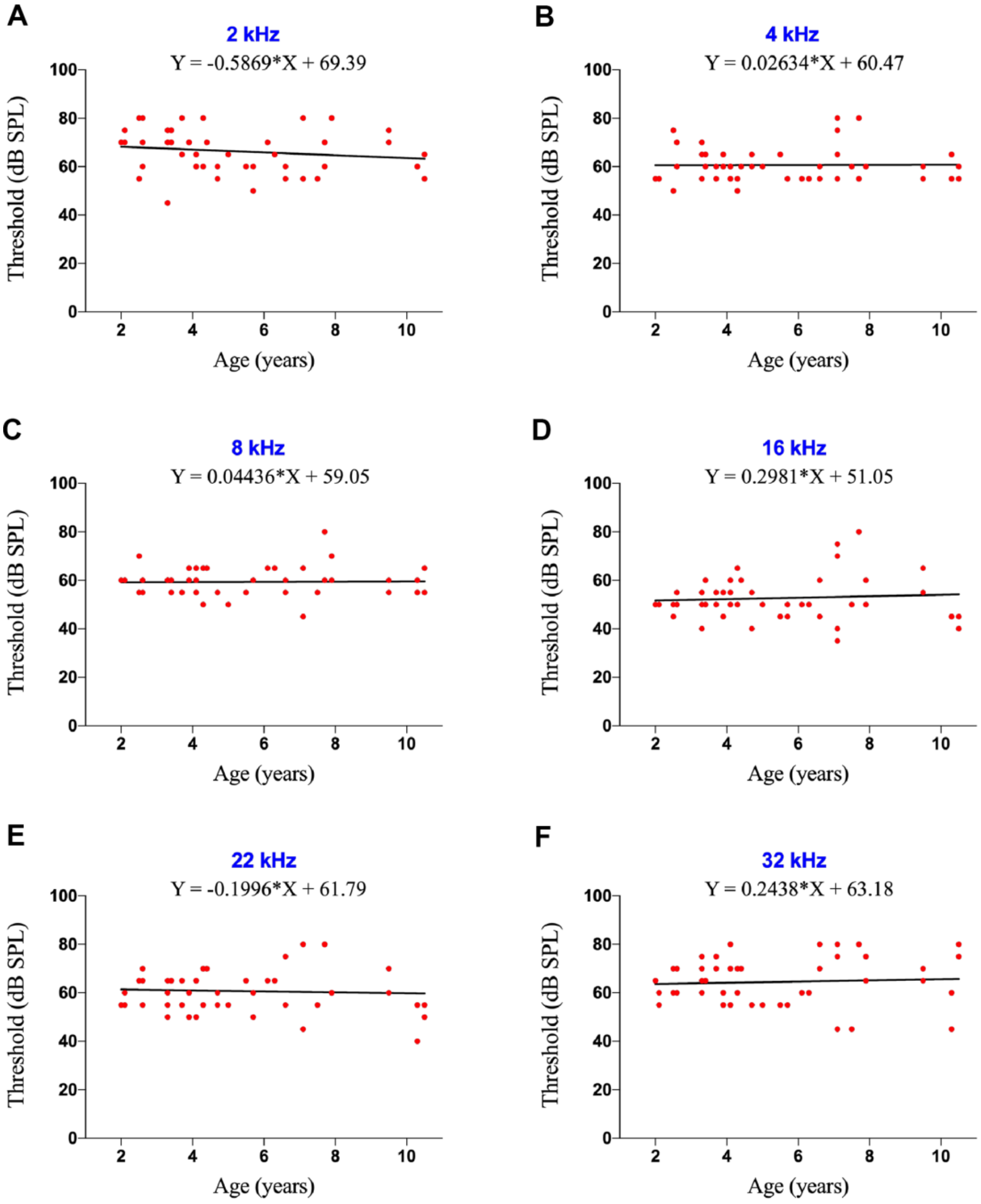

Supplementary Figure 3. Effects of age on DPOAE thresholds in marmosets. (**A**–**F**) Scatter plots and corresponding regression lines and regression equations for the relationships between DPOAE thresholds (dB SPL) in 51 ears of 36 marmosets and age (years) at frequencies of 2 kHz (**A**; R-squared linear=0.02534, P=0.2746); 4 kHz (**B**; R-squared linear=0.00009, P=0.9489); 8 kHz (**C**; R-squared linear=0.00032, P=0.9023); 16 kHz (**D**; R-squared linear=0.00591, P=0.5996); 22 kHz (**E**; R-squared linear=0.00285, P=0.7159); and 32 kHz (**F**; R-squared linear=0.00366, P=0.6796).
